# Dissociating between object affordances and spatial compatibility effects using early response components

**DOI:** 10.3389/fpsyg.2013.00591

**Published:** 2013-09-04

**Authors:** Meytal Wilf, Nicholas P. Holmes, Isabella Schwartz, Tamar R. Makin

**Affiliations:** ^1^Neurobiology Department, Hebrew University of JerusalemJerusalem, Israel; ^2^Department of Neurobiology, Weizmann Institute of ScienceRehovot, Israel; ^3^Centre for Integrative Neuroscience and Neurodynamics, School of Psychology and Clinical Language Sciences, University of ReadingReading, UK; ^4^Rehabilitation Department, Hadassah Medical CenterJerusalem, Israel; ^5^FMRIB Centre, Nuffield Department of Clinical Neuroscience, Oxford UniversityOxford, UK

**Keywords:** affordance, amputees, EMG, hand, stimulus-response

## Abstract

Perception and action are tightly linked: objects may be perceived not only in terms of visual features, but also in terms of possibilities for action. Previous studies showed that when a centrally located object has a salient graspable feature (e.g., a handle), it facilitates motor responses corresponding with the feature's position. However, such so-called affordance effects have been criticized as resulting from spatial compatibility effects, due to the visual asymmetry created by the graspable feature, irrespective of any affordances. In order to dissociate between affordance and spatial compatibility effects, we asked participants to perform a simple reaction-time task to typically graspable and non-graspable objects with similar visual features (e.g., lollipop and stop sign). Responses were measured using either electromyography (EMG) on proximal arm muscles during reaching-like movements, or with finger key-presses. In both EMG and button press measurements, participants responded faster when the object was either presented in the same location as the responding hand, or was affordable, resulting in significant and independent spatial compatibility and affordance effects, but no interaction. Furthermore, while the spatial compatibility effect was present from the earliest stages of movement preparation and throughout the different stages of movement execution, the affordance effect was restricted to the early stages of movement execution. Finally, we tested a small group of unilateral arm amputees using EMG, and found residual spatial compatibility but no affordance, suggesting that spatial compatibility effects do not necessarily rely on individuals' available affordances. Our results show dissociation between affordance and spatial compatibility effects, and suggest that rather than evoking the specific motor action most suitable for interaction with the viewed object, graspable objects prompt the motor system in a general, body-part independent fashion.

## Introduction

The idea that an object is perceived not only by its visual features, but also by the potential motor actions it affords (object affordances) has captured the attention and imagination of both scientists (e.g., Tucker and Ellis, 1998, 2004) and philosophers (Gibson, 1979). In support of this notion, a series of behavioral studies have shown that when a centrally located object has a salient graspable feature (e.g., a handle), it facilitates motor responses corresponding with the feature position (the “affordance effect”; Tucker and Ellis, 1998): When participants were asked to perform simple key-press responses with both hands, the response time of the hand most suited to perform a reach-and-grasp movement toward the object handle was speeded. Importantly, affordance effects occurred even when the objects themselves were irrelevant to the task performed by the participants (Phillips and Ward, 2002). Furthermore, objects that facilitate different kinds of grasping (e.g., power grip or precision grip), prime different motor actions accordingly (“micro affordances”; Ellis and Tucker, 2000; Tucker and Ellis, 2004). This line of evidence led researchers to conclude that object affordances automatically activate codes for actions appropriate for the utilization of that object, even when these responses are irrelevant.

However, it might be argued that the object's graspable feature draws attention to that location, thus facilitating responses made by the corresponding hand through a general spatial compatibility effect (Simon and Rudell, 1967). In recent years a controversy has developed around whether the affordance effect is a special case of spatial compatibility, or is in fact dissociated from stimulus-response compatibility effects. A few studies have tried to tease apart the two phenomena, with mixed results. For example Symes et al. (2005) simultaneously manipulated the spatial location of objects and the orientation of their handles, and found that each produced a distinct compatibility effect. These results were taken as an indication that affordance effects and the Simon effect are independent (see also Riggio et al., 2008; Pellicano et al., 2010). However, it is still possible that these results reflected two orthogonal compatibility effects. A similar confound may exist for micro-affordances, which have not been tested against non-graspable objects with similar shapes and sizes.

Other studies have found a tight link between the affordance and spatial compatibility effects (Anderson et al., 2002; Cho and Proctor, 2010; Kostov and Janyan, 2012). Anderson et al. (2002) compared speeded responses to drawings of graspable (scissors) and non-graspable (clocks) objects. They found that the fastest responses tended to arise on the side of the salient feature (handles or dials, respectively) regardless of the afforded motor actions on the object. They therefore concluded that the visual asymmetry of the target object creates an attentional shift leading to the affordance effect. One potential shortcoming of this study is that the authors used schematic line drawings that would not necessarily activate a motor response. As the affordance effect is theorized to be driven by an automatic and ecological motor response, there is a need to measure it with more naturalistic stimuli. Moreover, most experimental paradigms so far have not accounted for differences in salient asymmetrical visual features (e.g., handles) between experimental conditions.

In the present work, we used electromyography (EMG) to study early response patterns emerging in the proximal muscles of the arm during reaching-like movements cued by object images. The use of EMG provides an opportunity to gain insight into the temporal patterns associated with stimulus-response effects, by measuring the timing of different aspects of the movement, particularly the early and late components of muscle responses. In a second experiment, we applied the same experimental design using button-press responses. To dissociate spatial compatibility effects, affordance effects, and to assess their potential interaction, we had participants respond to images of typically graspable and non-graspable objects, presented either on the same or the opposite side to their responding limb. Each graspable object image was paired with a non-graspable object with similar asymmetry, thus accounting for the potential saliency of the handle. If graspable object images facilitate the motor system irrespective of spatial compatibility, then we should find a significant affordance effect (faster reaction times for graspable objects than for non-graspable objects) even while accounting for similarity in object asymmetry and position across conditions. Furthermore, if the affordance effect facilitates a specific motor action, we would expect a larger affordance effect when the object image appears on the same side as the hand most suitable to perform the grasping movement afforded by the object, reflected in an interaction between spatial compatibility and affordance.

A different approach that could help tease apart the potential action component from the perceptual one is that of testing affordance and spatial compatibility effects in populations with altered motor abilities. Unilateral arm amputees are a particularly interesting population in this context, as their disability results in lateralized limb-use, leading to spatially-asymmetrical interactions with objects in their environment. Indeed, we have recently shown that amputees exhibit distorted visuospatial representation of near space, such that they tend to over-represent distances on their intact side, compared to their amputated side (Makin et al., 2010). Based on this finding, we might expect to find modulated spatial compatibility and manipulability effects. By contrast, we have recently demonstrated a maintained representation of the phantom hand in the sensorimotor cortex of (acquired) amputees, as found during volitional phantom hand movements (Makin et al., 2013). This finding may suggest preserved stimulus-response compatibilities in amputees. To examine these hypotheses we conducted a third experiment where we recorded EMG measures during reaching-like movements in a group of individuals with a unilateral upper limb absence (here called “amputees”). This population also allowed us to explore the importance of recent interactions with objects on both affordance and spatial compatibility effects.

## Methods

### Participants

A total of 22 intact participants were recruited to the study. Ten participants took part in the EMG experiment (mean ± SD age 27 ± 4 years, all right handed). A total of 18 participants took part in the button press experiment (mean ± SD age 25 ± 3 years, 15 right handed), of whom 6 participated in the EMG experiment prior to the button press experiment. In addition, nine participants with upper limb amputation (mean ± SD age 44 ± 4 years, 4 with absent left hand, 4 with congenital deficiency, as determined by self-report, see Table 1) participated in a further EMG study. The handedness of intact participants was assessed using the 20-item version of the Edinburgh questionnaire (Oldfield, 1971). All procedures were approved by the Hadassah Medical Center Ethics Committee, and participants gave written informed consent prior to the experimental sessions.

**Table 1:** **Detailed information on amputee participants**.

**Amputee**	**Age**	**Years since amp.**	**Amp. hand (dominant hand?)**	**Amp. degree**	**Prosthetic/Frequency of use (0–5)**	**Phantom pain**	**Phantom limb sensation**	**Comp effect size (ms)**	**Afford effect size (ms)**
A01	31	31 (cong)	L(n/a)	Below elbow	Functional/4	Never	Never	−10.4	6.5
A02	50	50 (cong)	R(n/a)	Below elbow	Functional/4	Never	Never	6.8	−61.6
A03	40	18	L(No)	Below elbow	Cosmetic/5	Daily	Daily	−9.1	−38.8
A04	44	44 (cong)	R(n/a)	Below elbow	Cosmetic/4	Never	Never	25.7	8.1
A05	58	39	R(No)	Above elbow	Mechanic/5	Often	Daily	82.5	−38.7
A06	51	33	L(No)	Above hand	Mioelectric/5	Rarely	Daily	2.8	33.1
A07	51	36	L(Yes)	Through elbow	Mechanic/5	Daily	Daily	15.1	−17.4
A08	51	29	R(Yes)	Below elbow	No/0	Never	Never	69.6	−7.6
A09	25	25 (cong)	R(n/a)	Above hand	Cosmetic/5	Never	Never	6.9	26.8

Frequency of use of residual arm was assessed using a questionnaire [0—never, 1—rarely, 2—occasionally, 3—daily (<4 h), 4—daily (4–8 h), 5—daily (>8 h)]. None of the congenital amputees had residual fingers. Amp., amputation; L, left; R, right; cong, congenital amputee (i.e., amilian; self-report); n/a, not applicable; comp, spatially compatibility; afford, affordance. Spatially compatibility effect size = incompatible - compatible; Affordance effect size = non-graspable - graspable.

### Stimuli and experimental design

**Figure 1:**
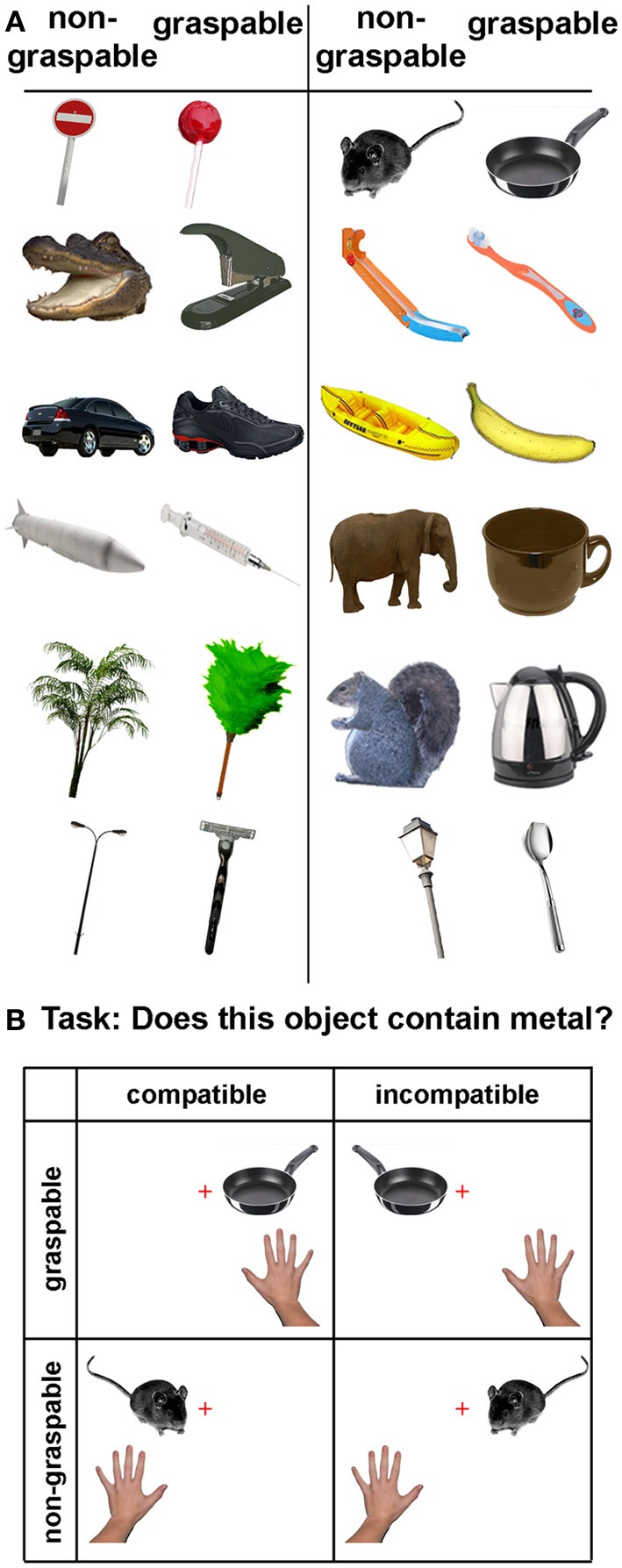
**Stimuli and experimental design. (A)** The object images used in the experiment. Each graspable object had a non-graspable counterpart, with similar asymmetrical features. Images were projected on a large screen, such that each image was presented near the left or right shoulder of the participants. **(B)** Experimental design. A 2 × 2 factorial design was used, with factors: graspability (graspable, non-graspable), and spatial compatibility (compatible, incompatible). The resulting four trial types are demonstrated, using one pair of objects. The hand illustrates the limb that will result in a correct response, and the cross illustrates the fixation point (in this example, subjects were asked to respond to metal objects with their right arm). Stimulus-response mapping was counterbalanced between subjects.

Stimuli consisted of 24 color pictures of everyday objects. The pictures were of typically graspable and non-graspable objects. Each graspable image had a matching non-graspable counterpart with similar visual features and size (see Figure 1A). Half of both the graspable and non-graspable objects contained metal. The experiment was conducted in a darkened room. Stimuli were controlled using the Presentation® software (Neurobehavioral Systems, Inc.) and projected on a large screen (163 × 203 cm), such that the image size was about 20 × 20 cm. The images appeared on either the left or the right lower side of the screen (28 cm from the center), at the subject's shoulder height. Trials were presented in a randomized order to avoid order related biases. Each image was presented for 300 ms, with 1700 ms intervals between trials, giving a total of 2000 ms for each trial. The graspable feature (e.g., the handle), or its visual homologue in non-graspable objects, always corresponded to the side of presentation (i.e., when the cup appeared on the right side its handle was oriented to the right as well).

Participants sat 50 cm in front of the center of the screen, within reaching distance, and were asked to keep their gaze on a red fixation cross, which appeared throughout the experiment. The participants performed a task relating to a stimulus feature unrelated to the graspability of the object—they were required to determine whether the object presented contained metal or not, by performing a speeded discrimination response with their left and right hands or arms (see Figure 1B). This task was chosen to ensure that the participants processed the image content, as studies have shown only an in-depth processing of the object is likely to induce an affordance effect (Symes et al., 2005; Pellicano et al., 2010). Stimulus-response mapping (left for metal, right for non-metal, and vice versa) was counterbalanced between subjects.

The responses were given either through button presses on a standard keyboard with the left or right index finger (“A” key or “num 6” key, respectively), or by lifting the arm to perform a reach-like movement toward the screen with the left or right arm, measured with EMG over the middle deltoid muscle. Participants were asked to raise the arm to shoulder height, such that the left/right hand (if available) was touching the left/right side of the screen (respectively) where the object images had been projected. To ensure that the participants were familiar with all the stimuli and were able to respond correctly, each session began with a slow presentation of the objects containing metal in the center of the screen, followed by the objects not containing metal. This was followed by a short training period (using two buttons), in which each object image was presented for 700 ms on either the left or the right side of the screen, and participants had to respond as quickly as they could. Following each trial in the training session, feedback was provided for both the accuracy and speed of the response.

After the training, participants performed two sessions where feedback was not provided. Each stimulus appeared 4 times (twice on the right and twice on the left side), with a total of 96 trials in each session. The amputee participants performed shorter sessions, with a total of 48 trials each.

### EMG recording and preprocessing

Adhesive disposable surface electrodes were placed over the right and left middle deltoid muscles in a belly-to-tendon fashion, with a reference electrode placed on the collar bone. EMG recording was triggered by Presentation software at the onset of each image presentation, using a sample rate of 2000 Hz. The signal was digitized using LabVIEW® and data were analyzed using custom scripts (available from the authors) in Matlab (MathWorks, Natick, MA). Offline, the data were segmented into 2000 ms epochs, baseline corrected, bandpass filtered with a dual-pass 4th order Butterworth filter (25–250 Hz), rectified, then low-pass filtered (<250 Hz). The baseline was defined as the first 100 ms of each trial (a voluntary EMG response typically has more than 120 ms latency; see Pruszynski et al., 2008). Single-sample “spike” artifacts were removed by interpolation. EMG data were analyzed by extracting a number of parameters: (1) the onset of voluntary EMG activity (“EMG onset”), defined as the first time-point after the baseline period (100 ms) for which the following 10 ms had a mean EMG activity greater than 3.09 standard deviations above the baseline mean EMG activity (i.e., where *p* < 0.001) (see Hodges and Bui, 1996). These parameters were chosen primarily for their robustness in discarding small spikes that were not followed by a full EMG response. (2) The latency of the maximum amplitude of the response (“EMG max”). This measure was chosen as a landmark in the reaching movement, due to its high correlation with button press reaction times, while measured from the same muscle (Figure S1). This measurement represents a later component of the movement.

### EMG analysis

In order to determine which of the two arms (left or right) was the responding arm, the maximum amplitude of each of the two EMG channels (left and right arms) was logged. The maximum amplitude values from each arm were normalized by dividing each value by the mean maximum amplitude across all trials with that arm. In each trial, the two normalized maximum amplitudes were compared. The arm more active in the trial (i.e., showing the highest normalized maximum amplitude) was defined as the responding arm, and its onset and maximum latencies were taken as reaction times (RTs). The onset of the EMG responses was expected to capture effects at the very early stages of the movement, reflecting sensory (and more automated) processing, with respect to later, more cognitive influences on motor execution (Lacouture and Cousineau, 2008). The maximum of the EMG response, was expected to reflect a later stage of motor response, corresponding to a button press (see Figure S1).

**Figure 2:**
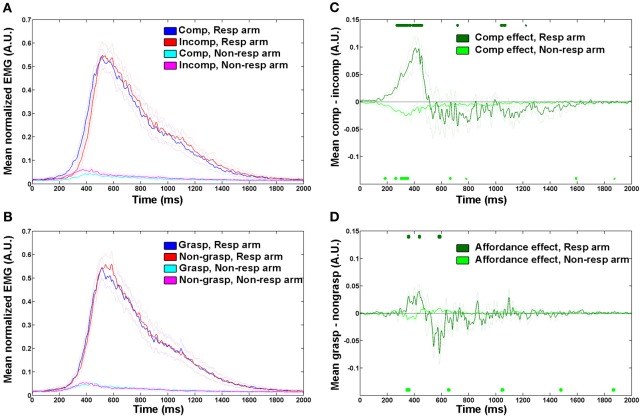
**Mean EMG traces showing spatial compatibility and affordance effects. (A,B)** Mean normalized EMG trace across all trials and participants in each condition in responding and non-responding arm for **(A)** spatial compatibility effect; **(B)** affordance effect. Dotted lines denote between-participant variability (SEMs). Time zero on the x-axis represents the stimulus presentation onset. **(C,D)** effect sizes in the responding- and non-responding arms for **(C)** spatial compatibility effect, calculated by (compatible—incompatible) mean response, **(D)** affordance effect, calculated by (graspable—non-graspable) mean response. Dotted lines denote between participants variability (SEMs). Circles on the top and bottom of plot indicate time points significantly different from baseline (*p* < 0.01, two-tailed). Comp, spatially compatible; incomp, spatially incompatible; resp, responding; grasp, graspable.

In order to display the mean EMG signal in each of the experimental factors (spatial compatibility and affordance, Figures 2A,B), the following additional steps were taken: Data were normalized to the mean of the maximum EMG of each of the participant's muscles to reduce between-participant and between-arm variability, and a further 50 Hz low-pass filter was used (note that this was done for visualization purposes only). Data from correct trials were sorted into 4 conditions for each of the two experimental factors: spatial compatibility (compatible vs. incompatible) or graspability (graspable vs. non-graspable) and for each arm (responding vs. non-responding). The mean EMG signal (across trials per condition then across participants) was plotted for each condition.

To plot the spatial compatibility and affordance effect sizes, the differences between the means of factor levels (compatible vs. incompatible and graspable vs. non-graspable) were calculated for each participant separately for the responding arm and the non-responding arms. Then the mean of all participants was plotted and statistical tests were performed to find time points significantly different from baseline (*p* < 0.01, two tailed) in each curve.

### “Twitch” analysis

In order to identify an early and automatic component of the motor responses, twitches were defined as follows: a significant elevation in EMG signal above baseline in the incorrect hand (i.e., the hand that would yield an incorrect response), which occurred prior to the correct hand response onset. The same onset criteria as “EMG onset” were used to determine EMG “twitches.” The percentage of twitches in each condition was calculated as the percentage of trials in which twitches were detected, out of the total number of trials in each condition (including error trials). Twitch data of one participant were lost due to a technical error.

### Statistical analysis of response latencies and reaction times

The EMG parameters and button press reaction times (RTs) were analyzed using Excel and Matlab. Each trial was assigned to one of four conditions, based on two factors: spatial compatibility (between the stimulus position and the responding hand, compatible vs. incompatible) and affordance (graspable vs. non-graspable, see Figure 1B). RTs on incorrect trials were discarded, as well as RTs longer than 1500 ms. RTs of each condition were averaged within each subject, and a Two-Way repeated measures ANOVA was performed using Matlab (spatial compatibility × affordance). Due to the small sample sizes used in this study, significant results of each test were further validated using the Wilcoxon rank sign test, which yielded similar results to the ANOVA main effects. Wilcoxon tests were also performed to test for compatibility and affordance effects in the error rates (calculated as percentage of incorrect responses out of the total number of trials in each condition). Effect sizes were calculated both by differences between the means (spatially incompatible-compatible and non-graspable-graspable), and as Cohen's d using online software (http://www.cognitiveflexibility.org/effectsize/).

For the amputee group, an additional factor of the amputated vs. intact arm was tested against the spatial compatibility and manipulability effects, resulting in a 3-way repeated measures ANOVA (spatial compatibility × affordance × arm). To account for variance in performance, resulting from amputation-related visuospatial perceptual biases, we used individuals' point of subjective equality (PSE; Makin et al., 2010) as a covariate in a further 3-way ANCOVA. The PSE measure captures lateral asymmetry in visuospatial representation of near space, as measured in a “landmark” task (for further details see Makin et al., 2010).

### Object familiarity analysis

In a *post-hoc* analysis, an observer, unaware of the study purposes, was asked to name each of the objects used in the study. Each object was then checked for prevalence in a large word and phrases corpus (google books Ngram Viewer, Michel et al., 2011). For each word the percentage of its appearance out of all the phrases of the same length in the corpus was calculated (in the Hebrew database between the years 2000 and 2008). Then the mean prevalence in each object group (graspable and non-graspable) was calculated and a two-tailed *t*-test was performed.

## Results

### EMG of reaching-like movements experiment

We first explored the spatial compatibility and affordance effects by plotting the mean EMG response of correct trials in the spatially compatible and incompatible conditions, for both the responding arm and the non-responding arm (Figure 2A). The response dynamics in this reach-like movement were of a quick elevation and a slower decay of the EMG signal, until it returned back to baseline at around 1800 ms from trial onset. In accordance with previous studies, we saw an earlier onset of the motor response for the compatible condition compared with the incompatible one. To better visualize the effect, we plotted the mean difference between spatially compatible and incompatible conditions in each time point of the trial. This allowed us to identify a clear increase in EMG signal in the spatially compatible condition in the responding arm around 250–450 ms from trial onset, deriving from an earlier rise in the signal (Figure 2C). A homologous effect was observed in the non-responding arm, where the incompatible condition had a higher amplitude and earlier onset. This could imply an arousal of the hand closest to the stimulus, even in the absence of a full motor response.

For the affordance effect we observed a smaller difference between graspable and non-graspable conditions (Figure 2B). The response to the graspable condition preceded that of the non-graspable condition at the very early stages of the response, at around 350 ms from trial onset (Figure 2D).

**Figure 3:**
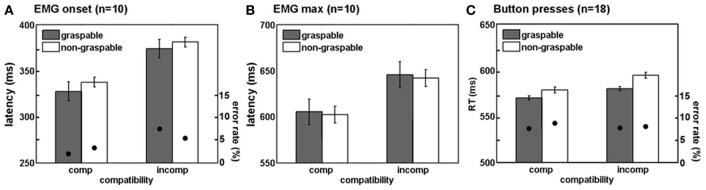
**Reaction time and EMG latency results show independent spatial compatibility and affordance effects**. Mean reaction times or EMG latencies (bars, left scale) and error rates (black circles, right scale) for intact participants for the four trial types comprising the factorial analysis (see Figure 1B), during onset of electromyography (EMG) response **(A)**, maximum amplitude of EMG response **(B)**, and button press responses **(C)**. Comp, spatially compatible; incomp, spatially incompatible. Error bars indicate confidence interval for means, while taking into account the within-participant design (Cousineau, 2005; Morey, 2008).

To quantify these observations and determine the relative contribution of affordance and spatial compatibility effects at early vs. later stages of the movement, we measured EMG onset and maximum amplitude latencies in each individual trial. When considering the onset of the voluntary EMG response (Figure 3A), we found significant spatial compatibility and affordance effects [*F*_(1, 9)_ = 11.46, *p* = 0.008 and *F*_(1, 9)_ = 7.11, *p* = 0.026, respectively] with large effect sizes (45 ± 13 ms, Cohen's *d* = 1.6 for spatial compatibility, and 9 ± 3 ms, Cohen's *d* = 0.9 for affordance), and no interaction [*F*_(1, 9)_ = 0.06, *p* = 0.815]. These results suggest that both spatial compatibility and affordance effects are present at the early component of the movement. In the later stage of response, when the EMG response is maximal (Figure 3B), the spatial compatibility effect was still evident [*F*_(1, 9)_ = 5.32, *p* = 0.046] albeit smaller (40 ± 17 ms, Cohen's *d* = 0.8). The affordance effect was gone [*F*_(1, 9)_ = 0.41, *p* = 0.430], and no significant interaction between spatial compatibility and affordance was found [*F*_(1, 9)_ = 0.01, *p* = 0.974].

This might imply that while the spatial compatibility effect has an extended time-window, a more transient influence of affordance restricts it to the earlier stages of the motor act. Alternatively, it might be possible that since the values of maximum EMG response are more variable, this measure is less sensitive to the small affordance effect. To address this potential confound, we calculated the coefficient of variation (*CV*) for each participant (SD/mean calculated for both onset and maximum latencies). This analysis revealed that the EMG max was less variable, compared with the EMG onset (*CV*_onset_ = 0.31 ± 0.006; *CV*_max_ = 0.22 ± 0.003, *p* = 0.001 in a 2-tailed *t*-test). Moreover, the mean affordance effect, as displayed in Figure 2D, suggests that by the time the EMG response is maximal (at around 600 ms), the affordance effect is abolished (as reflected in higher EMG amplitude to the non-graspable conditions).

### Button press response experiment

Next, we studied the spatial compatibility and affordance effects using the same set of stimuli but with a more conventional button press response. Using a larger group of 18 participants, we identified significant compatibility and affordance effects [*F*_(1, 17)_ = 8.32, *p* = 0.01 and *F*_(1, 17)_ = 7.27, *p* = 0.015, respectively; Figure 3C], again with no interaction [*F*_(1, 17)_ = 1.25, *p* = 0.27]. However, effect sizes were smaller (13 ± 4 ms, Cohen's *d* = 0.7 for spatial compatibility and 12 ± 4 ms, *d* = 0.65 for affordance) suggesting that the traditional button response approach is less sensitive in capturing the early stages of the response (as shown using EMG onset), and therefore requires a larger sample size to reveal both effects. When examining a subgroup of 12 participants that performed the button press experiment but not the EMG experiment prior to it, the effects were mostly retained [*F*_(1, 11)_ = 4.16, *p* = 0.06 for spatial compatibility effect, and *F*_(1, 11)_ = 6.45, *p* = 0.027 for affordance effect].

### “Twitch” results

**Figure 4:**
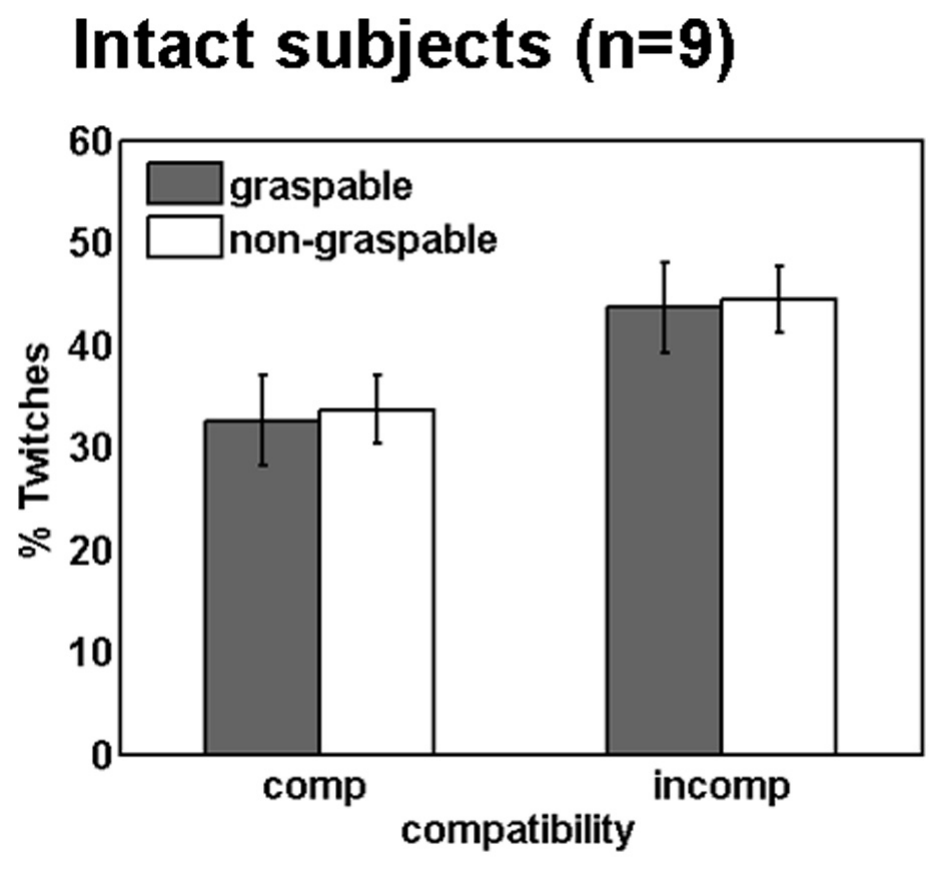
**Spatial compatibility effects during movement preparation in intact participants**. Mean percentage of twitches (i.e., EMG activity in the hand irrelevant for response, see methods) in intact subjects. Annotations are as in Figure 3.

To study motor responses to the visual stimulus that are potentially involuntary, we assessed EMG activity prior to the correct response in the incorrect hand (“twitches”). During incompatible trials, participants are required to suppress an early response with the arm that is spatially compatible with the stimulus, in order to respond correctly. This process might account to some extent for the delayed responses in spatially incompatible (compared to compatible) trials. Moreover, we were interested to see whether graspable object images would induce more twitches than non-graspable images, due to their motor arousal effect. In the present study, participants produced more twitches in spatially incompatible trials, as compared to compatible trials. In other words, the non-responding arm was more active when the objects were presented next to it, resulting in a trend toward a significant spatial compatibility effect [*F*_(1, 8)_ = 3.82, *p* = 0.08; Figure 4]. However, similar trends were not found for the affordance effect [*F*_(1, 8)_ = 0.06, *p* = 0.8], or for an interaction with the affordance effect [*F*_(1, 8)_ = 0.11, *p* = 0.74].

### EMG of reaching-like movements in amputees

**Figure 5:**
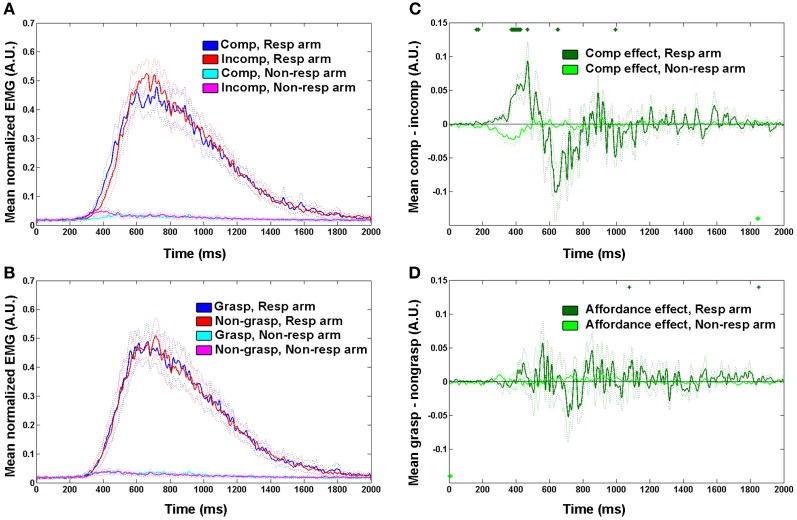
**Mean EMG traces for (A) spatial compatibility effect; (B) affordance effect**. Effect sizes for **(C)** spatial compatibility effect; **(D)** affordance effect. Annotations are as in Figure 2.

**Figure 6:**
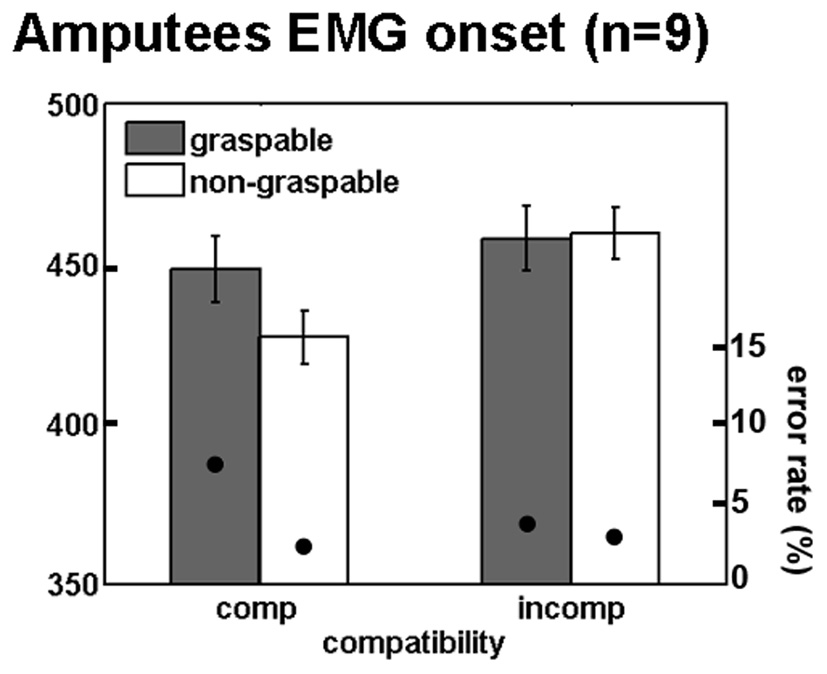
**Maintained spatial compatibility effect in unilateral upper limb amputees**. Mean reaction times and error rates for amputees for the four trial types comprising the factorial analysis, during onset of EMG response. Annotations are as in Figure 3.

We tested our experimental paradigm on nine upper limb amputees using EMG of the deltoid muscles while they were performing reaching responses with their intact and residual arms. Since the effect size was greatest for EMG onset in the intact participants dataset, for the amputee group we focused our analysis on EMG onset latencies. No differences in onset latencies were found between movements executed with the intact arm and the residual arm (*p* = 0.77), and no interaction was found between the responding arm (intact vs. residual) and each of the other effects [*F*_(1, 7)_ = 0.8, *p* = 0.39 and *F*_(1, 7)_ = 1.35, *p* = 0.27, for spatial compatibility and affordance, respectively]. We therefore plotted the normalized mean EMG response, averaged across both arms, in a similar manner to the intact participants (Figure 5). The response dynamics were generally similar to those observed in intact participants (although relatively delayed), with a rapid rise of EMG signal and a slower decay toward baseline (Figures 5A,B). When considering the effect of spatial compatibility, the effect was restricted to the early stages of the movement (around 400 ms; Figure 5C), with the effect reversing as the amplitude for the incompatible condition reached its maximum (Figures 5A,C). In contrast, the affordance effect was completely absent in the amputee's data (Figures 5B,D). When applying a Three-Way ANOVA to the onset latencies we found a trend toward a spatial compatibility effect [*F*_(1, 7)_ = 3.6, *p* = 0.09, 21 ± 4 ms, Cohen's *d* = 0.72], no affordance effect [*F*_(1, 7)_ = 0.89, *p* = 0.37, −10 ± 4 ms, Cohen's *d* = 0.37] and no interaction [*F*_(1, 7)_ = 0.78, *p* = 0.39; Figure 6]. When applying a 3-way ANCOVA, taking into account the spatial biases of each amputee participant (using individual PSE values as a covariate, see introduction and methods), we found a significant spatial compatibility effect [*F*_(1, 7)_ = 9.6, *p* = 0.017], as well as a significant interaction between the spatial compatibility effect and the spatial PSE [*F*_(1, 7)_ = 10.1, *p* = 0.016]. No other significant main effects or interactions were found (*p* > 0.18).

### Speed-accuracy trade-offs

In order to account for potential confounds resulting from speed-accuracy trade-offs, effects of spatial compatibility and affordance were also tested on the error rates in each of the experiments. None of the results were significant (*p* > 0.15), with the exception of a trend toward a significant spatial compatibility effect in the intact participants EMG data (*p* = 0.06), showing more errors were performed in the spatially incompatible condition.

### Accounting for differences between images

It could be argued that the affordance effect we found in the intact group was due to other parameters differentiating the object groups (like familiarity). To account for this potential confound we identified the frequency of appearance of the name of each object in a large word and phrases corpus (Michel et al., 2011). We found the mean frequency of appearance in the corpus was 4*e*^−4^ ± 1*e*^−4^ for graspable and 9*e*^−4^ ± 4*e*^−4^ for non-graspable objects, with no significant difference between the object groups (*p* = 0.22).

## Discussion

Using an ecological setup, with naturalistic images and whole arm reaching-like responses, we present new and converging evidence for the existence of an affordance effect independently of spatial compatibility. We found that in intact participants, responses to graspable objects were faster than to non-graspable objects, independently of spatial compatibility. These results were replicated both with button press responses, and when measuring the onset of a reach-like movement using EMG. The prevalent account of affordances, based on speeded RTs for graspable objects oriented toward the responding hand, suggests lateralized facilitation of the hand toward which the central object's handle is oriented (Tucker and Ellis, 1998; Phillips and Ward, 2002; McBride et al., 2012). When considering responses for graspable objects only, we found that RTs were speeded for the spatially compatible hand. However, RTs for non-graspable objects showed similar compatibility effects, resulting in no significant interaction between object position (spatially compatible vs. incompatible) and object affordance (graspable vs. non-graspable). The fact that we found no interaction but two independent main effects suggests that graspable objects induce general arousal of the motor system, rather than evoking a specific potential motor action, based on the best motor plan afforded by the objects' position (as suggested in Gibson, 1979; Makris et al., 2011). Our results therefore support the view advocated by Cho and Proctor (2010) and Kostov and Janyan (2012) that the affordance effects, as shown for lateralized object positions (Tucker and Ellis, 1998; Phillips and Ward, 2002; McBride et al., 2012) may have been partly due to orientation of spatial attention toward the handle, leading to a classic spatial compatibility effect.

While we demonstrated the independent presence of the affordance and the spatial compatibility effects using the “classical” button press paradigm, both effects were more evident (as demonstrated by larger effect sizes) when responses were measured using a more ecological motor response (EMG recordings of reach-like movements). The EMG recordings also enabled us to monitor more closely the time course of each of the two effects. We found that the spatial compatibility effect was present from the earliest stages of movement preparation (“twitches”), through early stages of movement execution (EMG onset) and to the late stages of the response (EMG max). The affordance effect, on the other hand, was relatively short-lived, as it was restricted to the early stages of movement execution (EMG onset). A recent study by McBride et al. (2012) reported speeded responses (using EMG recording of distal hand muscles) in the hand corresponding to the object handle position, however this study did not account for non-graspable objects. Our results extend these findings by providing further evidence for the existence of an affordance effect as an early and transient component of motor control.

The use of EMG also made it possible for us to look at early motor activity in the non-responding hand. We found that more twitches were made on spatially incompatible trials (i.e., when the stimulus was presented near the non-responding hand), resulting in a trend toward a significant compatibility effect. This trend is in line with the “activation suppression hypothesis,” which posits that in order to perform an incompatible response, it is necessary to first suppress compatible motor responses from the non-responding hand (Ridderinkhof, 2002). Similar results were observed by Burle et al. (2002), who measured EMG of distal muscles during a spatial compatibility task. The authors found that the prolonged RTs in incompatible trials corresponded to the existence of “twitches” in the wrong hand during the preliminary response stages of those trials. We found no evidence for early competition in response selection for graspable objects (compared with non-graspable objects). While null results should be interpreted with caution, this result might further support the notion that the affordance effect is not effector specific, because the graspability of the object did not provoke a competition between the two hands, but merely a speeding of response in the responding hand.

The spatial compatibility effect was also observed in the EMG responses of unilateral upper-limb amputees. Importantly, this effect was exposed only after accounting for the contribution of the visuospatial perceptual asymmetry that resulted from the amputation. This might imply that several independent processes may be influencing the amputees' performance, such that it is necessary to tease apart factors contributing to both maintained and altered visuospatial representations in order to interpret their behavior. A recent study showed that stimulus-response compatibility effects between two fingers of the intact hand are unaffected by amputation (Philip and Frey, 2013)—right finger responses were made more quickly to stimuli presented on the right side of the screen as compared to stimuli presented in the middle or the left of the screen, while left finger responses were quicker for stimuli presented on the left. This suggests that the spatial compatibility effects within the intact hand are maintained following amputation. Our results extend this notion, by demonstrating that bimanual response-selection mechanisms underlying spatial compatibility effect are still preserved to some extent, and do not exclusively depend on recent experience.

Research with special populations provides an exciting opportunity for teasing apart the affordance and spatial compatibility effects. For example, it has been shown that in healthy volunteers, but not in Parkinson's patients, a compatibility effect was enhanced by graspable stimuli (i.e., door handles; Poliakoff et al., 2007) (However, note that in this study graspable objects had been shown to result in longer RTs, compared to non-graspable objects (bars), making any further interpretation of the relationship between compatibility and affordance tenuous). While we found clear evidence for a compatibility effect, we could not find any traces of an affordance effect in amputees, or an interaction between affordance and spatial compatibility or responding hand. These results therefore support the notion that the affordance effect we identified does not depend on a lateralized action plan, as considered before (Tucker and Ellis, 1998). Rather than being body-part specific, object affordance may depend on the indiscrete functioning of the motor system, however more research is needed to carefully assess the affordance effect on amputees.

The ecological design that we used in this study raises several methodological confounds that might be worth considering. First, EMG recording from proximal muscles might be considered more relevant for reaching than for grasping movements. However, while reaching and grasping movement components have specialized neural mechanisms (Cavina-Pratesi et al., 2010a,b), previous research points at a tight dependence and coordination between those two types of movements. Perturbation of only one of the components affects the dynamics of the other (Paulignan et al., 1991a,b; Jeannerod, 1999), and designated brain regions support their coordination in a reach-to-grasp movement (Cavina-Pratesi et al., 2010b). For this reason, we expect to identify changes in proximal muscles associated with graspable object features. Indeed, the dissociation between the two image categories we used as stimuli more critically depends on their potential for execution of a grasping movement, rather than their reachability (while a car in itself is typically non-graspable, it is nevertheless reachable). Accordingly, we identified comparable affordance effects using both proximal muscles (EMG recordings) and distal muscles (button responses).

A second potential confound arises from the use of naturalistic stimuli, which are more susceptible to confounds deriving from unexpected differences between object groups. To reduce this confound, we attempted to span a relatively large range of stimuli, with similar familiarity. But other parameters may influence the results. For example, non-graspable objects are typically larger in real life than graspable objects. Previous studies have found that large objects typically show faster responses than small objects (though non-significant, Tucker and Ellis, 2004; Vainio et al., 2006), thus this confound probably cannot account for the results we report. Similarly, a significant proportion of the non-graspable stimuli were natural, rather than man-made. But given the judgment participants were making (whether the object contains metal), responses should, if anything, have been faster for the naturalistic images (which usually do not contain metal). We therefore believe that the effect of affordance was likely based on the dissociation of the two groups into graspable and non-graspable objects, although more careful categorization of the stimuli is necessary.

To conclude, using EMG recordings of proximal muscles we demonstrate earlier motor responses to graspable objects, irrespective of whether the responding arm is most suitable to perform a reaching movement toward that object. Our results therefore prompt a revisit of the classical definition of the affordance effect as “operation of intentions to act on already existing motor representations of the possible actions in a visual scene” (Tucker and Ellis, 1998). Instead, our results suggest that graspable objects activate the motor system in a general, body-part independent fashion.

### Conflict of interest statement

The authors declare that the research was conducted in the absence of any commercial or financial relationships that could be construed as a potential conflict of interest.

## Supplementary material

Figure S1 **Relationship between EMG parameters of the first dorsal interosseous (FDI) muscle and index-finger button press reaction time (RT)**. Participants were required to perform a simple button press with their index finger in response to a visual cue. EMG was recorded from the FDI during task performance. The same EMG analysis was performed as described in the main text. The plots here show the relationship between RTs recorded using a button press and between EMG max **(A)** and onset **(B)** in one representative participant. Correlation coefficients (Pearson's r) were averaged for each individual across two hand positions, and then across 10 participants **(C)**. On average, both latencies of EMG maximum (max time) and onset showed strong correspondence with RT values (*p* < 0.0001). The area under the EMG envelop (area) also showed a significant relationship with RT values (*p* < 0.05), although to a much reduced extent. EMG amplitude (mV) at maximum latency (max amp) and mean amplitude during EMG baseline (mean baseline) didn't show any significant relationship with button press RT. Based on these findings, EMG maximum and onset latencies have been identified as the most relevant EMG parameters as proxies for RT. Error bars show s.e.m. Data was taken from Makin et al. (2009), Experiment 1.
