# Supplementary material for: Dissociating between object affordances and spatial compatibility effects using early response components
Source: Front Psychol. 2013 Sep 4;4:591. doi: 10.3389/fpsyg.2013.00591 (PMC3761160; doi:10.3389/fpsyg.2013.00591)
Supplement: Figure S1 — Relationship between EMG parameters of the first dorsal interosseous (FDI) muscle and index-finger button press reaction time (RT). Participants were required to perform a simple button press with their index finger in response to a visual cue. EMG was recorded from the FDI during task performance. The same EMG analysis was performed as described in the main text. The plots here show the relationship between RTs recorded using a button press and between EMG max (A) and onset (B) in one representative participant. Correlation coefficients (Pearson's r) were averaged for each individual across two hand positions, and then across 10 participants (C). On average, both latencies of EMG maximum (max time) and onset showed strong correspondence with RT values (p < 0.0001). The area under the EMG envelop (area) also showed a significant relationship with RT values (p < 0.05), although to a much reduced extent. EMG amplitude (mV) at maximum latency (max amp) and mean amplitude during EMG baseline (mean baseline) didn't show any significant relationship with button press RT. Based on these findings, EMG maximum and onset latencies have been identified as the most relevant EMG parameters as proxies for RT. Error bars show s.e.m. Data was taken from Makin et al. (2009), Experiment 1. [file Presentation1.PDF]

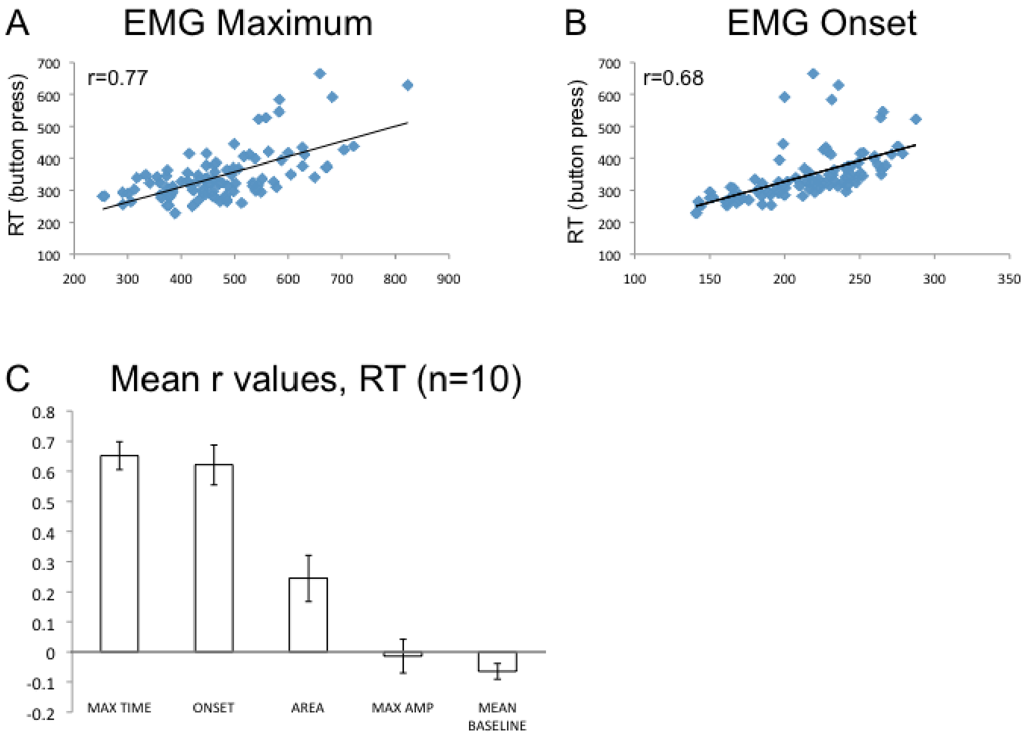

Figure S1. Relationship between EMG parameters of the first dorsal interosseous (FDI) muscle and index-finger button press reaction time (RT). Participants were required to perform a simple button press with their index finger in response to a visual cue. EMG was recorded from the FDI during task performance. The same EMG analysis was preformed as described in the main text. The plots here show the relationship between RTs recorded using a button press and between EMG max (A) and onset (B) in one representative participant. Correlation coefficients (pearson's r) were averaged for each individual across two hand positions, and then across 10 participants (C). On average, both latencies of EMG maximum (max time) and onset showed strong correspondence with RT values ($p<0.0001$). The area under the EMG envelop (area) also showed a significant relationship with RT values ($p<0.05$), although to a much reduced extent. EMG amplitude (mV) at maximum latency (max amp) and mean amplitude during EMG baseline (mean baseline) didn't show any significant relationship with button press RT. Based on these findings, EMG maximum and onset latencies have been identified as the most relevant EMG parameters as proxies for RT. Error bars show s.e.m. Data was taken from Makin et al., 2009, Experiment 1.
